# Generation of transgene-free human induced pluripotent stem cells from erythroblasts in feeder-free conditions

**DOI:** 10.1016/j.xpro.2022.101620

**Published:** 2022-08-17

**Authors:** Sylvain Perriot, Mathieu Canales, Amandine Mathias, Renaud Du Pasquier

**Affiliations:** 1 Laboratory of Neuroimmunology, Neuroscience Research Centre, Department of Clinical Neurosciences, Lausanne University Hospital (CHUV) and University of Lausanne, Lausanne, Switzerland; 2 Service of Neurology, Department of Clinical Neurosciences, Lausanne University Hospital (CHUV) and University of Lausanne, Lausanne, Switzerland

**Keywords:** Cell Biology, Cell culture, Cell isolation, Stem Cells, Biotechnology and bioengineering

## Abstract

This protocol describes the generation and characterization of human induced pluripotent stem cells (hiPSCs) from erythroblasts. A key difference with classical protocols is the reprogramming of erythroblasts from a simple blood draw as opposed to fibroblasts/keratinocytes, which requires a biopsy. Moreover, working with erythroblasts ensures that no recombination of the TCR/BCR genes occurs, as opposed to T cells and whole peripheral blood mononuclear cells-based approaches. Last, this approach uses non-integrative episomes ensuring no integration of transgenes into the hiPSCs genome.

For complete details on the use and execution of this protocol, please refer to Perriot et al. (2018).

## Highlights

• Generation of human iPSCs from only 10 mL of blood
• An approach that is completely feeder free and transgene free
• Quality controls following the guidelines edited by the European Bank for iPSCs (EBiSC)
• Absence of TCR/BCR genomic recombination attributed to erythroblast reprogramming

## Abstract (editor highlights)

Publisher’s note: Undertaking any experimental protocol requires adherence to local institutional guidelines for laboratory safety and ethics.

## Graphical abstract

**Figure:**
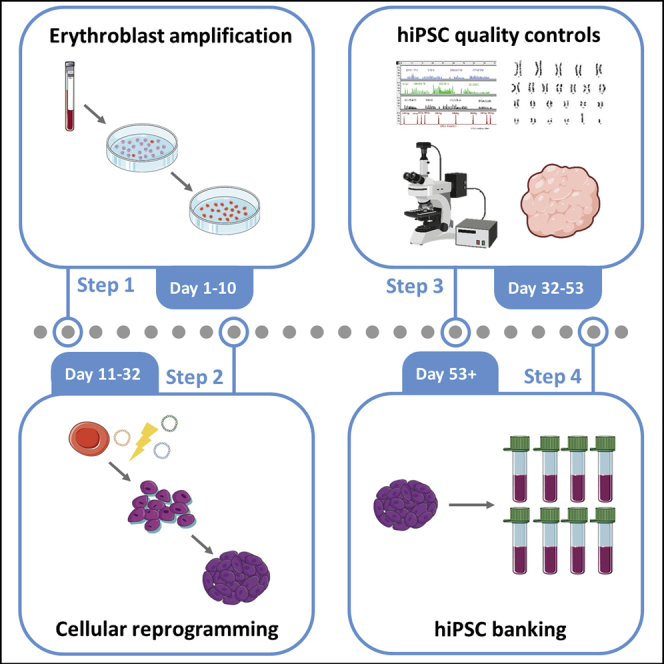

## Before you begin

The execution of this protocol requires prior blood draw and standard peripheral blood mononuclear cells (PBMCs) isolation. Only 10 mL of blood collected in EDTA tubes is required. This protocol is suitable for the reprogramming of erythroblasts from both adults and children (protocol tested in our hands on donors from 2 to 55 year-old without any difference in efficiency. The only difference observed is that erythroblasts from children are redder than from adults but this does not affect the efficiency of reprogramming).

Human erythroblasts, hiPSCs and differentiated cells were all cultured in a humidified 37°C incubator and 5% CO_2_.

Prior to beginning the reprogramming protocol, prepare media, solutions, and matrix-coated culture dishes. Once prepared, erythroblast medium can be kept at 4°C for 1 week while ReproTeSR and StemMACS human iPSC Brew media can be kept at 4°C for 2 weeks. All media should be brought to room temperature or 37°C prior to adding on the cells.

This protocol is optimized to be started on a Thursday to minimize cell handling during the weekends.

### Matrigel coating

**Timing: 1 h**

Matrigel should be kept under 10°C when handling to avoid polymerization. We recommend using pipet tips and tubes pre-cooled at 4°C for aliquoting and handling the Matrigel.

***Note:*** Aliquots are made at receipt of Matrigel to ease its use on daily routine. We prepare aliquots of 0.5 mg, 1 mg and 2 mg and store them at −20°C until use.

1. Thaw Matrigel at 4°C.
2. Dilute Matrigel in cold DMEM/F12 as per dilution factor mentioned on the datasheet (variable for each Matrigel lot, final concentration of 100 μg/mL).
3. Add the diluted Matrigel to the dish in order to cover the entire surface. In this protocol, we are using 10 cm^2^ dishes or wells which are coated with 1 mL of diluted Matrigel (10 μg/cm^2^).
4. Incubate at room temperature for 60 min. Coated dishes can be kept at 37°C for 3 days.
5. Plates are ready to use after removal of the Matrigel solution, there is no need for washing the plates before use.

### Institutional permissions

This study was performed according to the ethics protocol approved by our institutional review committee (approval CER-VD 2018-01622, Lausanne, Switzerland). All subjects enrolled or their legal representatives gave their written informed consent according to the study protocol.

## Key resources table

**Table:** 

REAGENT or RESOURCE	SOURCE	IDENTIFIER
**Antibodies**		

CD36-FITC 1 mL (1:50)	Miltenyi	130-095-470
CD71-PE 1 mL (1:100)	Miltenyi	130-091-728
CD71 Microbeads 2 mL	Miltenyi	130-046-201
anti-Sox2 mouse IgG (1:200)	Abcam	AB79351
anti-Tra-1-60 mouse IgG (1:200)	Millipore	MAB4360
anti-Oct4 mouse IgG (1:200)	SCB	sc-5279
anti-SMA mouse IgG (1:200)	Dako	M1110851
anti-Pax6 rabbit IgG (1:100)	BioLegend	PRB-278P
anti-AFP mouse IgG (1:100)	Sigma-Aldrich	A8452

**Biological samples**		

Human blood	Male or female donor, aged 2–55	N/A

**Chemicals, peptides, and recombinant proteins**		

StemSpan SFEM Medium	StemCell Technologies	09605
Ficoll-Paque PLUS	Cytiva	17144003
Human Erythropoietin	R&D Systems	287-TC-500
Human SCF	R&D Systems	255-SC-010
Human IGF-1	Miltenyi	130-093-885
Human IL-3	Miltenyi	13-093-908
Dexamethasone powder	Sigma-Aldrich	D4902-25MG
Cholesterol Lipid Concentrate 250×	Life Technologies	12531-018
Human CD34+ Nucleofector kit, 25 reactions	Lonza	VPA-1003
ReproTeSR medium	StemCell Technologies	5920
Matrigel® hESC-Qualified Matrix, ∗LDEV-Free	Corning	354277
StemMACS iPS-Brew XF, human	Miltenyi	130-104-368
StemMACS Y27632 in Solution	Miltenyi	130-106-538
ReLeSR	StemCell Technologies	5873
Cryostor CS10	StemCell Technologies	7930
Bovine serum albumin (BSA)	Sigma-Aldrich	A3912
EDTA solution 0.5 M	Sigma-Aldrich	03690
MycoAlertTM Mycoplasma Detection Kit	Lonza	LT07-318
PowerPlex® 16 System	Promega	DC6531
DMEM/F-12	Life Technologies	31331028
RPMI 1640 Medium	Life Technologies	61870010
Dimethyl sulfoxide (DMSO)	Sigma-Aldrich	D2650-100ML
Fetal bovine serum (FBS)	Sigma-Aldrich	F7524-500ML
Accutase	Life Technologies	A1110501
PBS (1×) without Ca Mg Phenol	Life Technologies	14190136
Paraformaldéhyde 16% EM Grade	Electron Microscopy Sciences	15710
Cell lifter	Corning	3008-COR

**Recombinant DNA**		

pCXLE-hOCT3/4-shp53-F	Okita et al. (2011)	Addgene 27077
pCXLE-hSK	Okita et al. (2011)	Addgene 27078
pCXLE-hUL	Okita et al. (2011)	Addgene 27080
pCXLE-EGFP	Okita et al. (2011)	Addgene 27082

**Oligonucleotides**		

EBNA1_forward	Perriot et al. (2018)	Table 2
EBNA1_reverse	Perriot et al. (2018)	Table 2
β-Actin_forward	Perriot et al. (2018)	Table 2
β-Actin_reverse	Perriot et al. (2018)	Table 2

**Other**		

Amaxa Nucleofector 2b	Lonza	AAB-1001

## Materials and equipment

Human erythropoietin, SCF, IGF-1 and IL-3 are all dissolved in sterile PBS with 0.5% BSA. Dexamethasone is dissolved in ethanol. Powders are dissolved, aliquoted and stored at −20°C as displayed in the table below. After thawing, these reagents can be kept for 10 days at +4°C. Avoid freezing-thawing cycles.

**Table:** 

Reagent	Stock concentration	Working concentration	Dilution factor	Storage
Erythropoietin	1000 unit/mL	2 unit/mL	1:500	−20°C
SCF	50 μg/mL	50 ng/mL	1:1′000	−20°C
IGF-1	40 μg/mL	40 ng/mL	1:1′000	−20°C
IL-3	10 μg/mL	10 ng/mL	1:1′000	−20°C
Dexamethasone	10 mM	1 μM	1:10′000	−20°C

**Table:** Erythroblast medium (to keep at 4°C for up to 1 week)

Reagent	Final concentration	Amount
StemSpan SFEM Medium		49.5 mL
Human SCF	50 ng/mL	50 μL
Human IGF-1	40 ng/mL	50 μL
Human IL-3	10 ng/mL	50 μL
Lipid cholesterol	1×	200 μL
Dexamethasone	1 μM	5 μL
Human Erythropoietin	2 U/mL	100 μL
**Total**		50 mL

**Table:** ReproTeSR medium (to keep at 4°C for up to 2 weeks)

Reagent	Final concentration	Amount
ReproTeSR™ Basal Medium		480 mL
ReproTeSR™ 25× Supplement	1×	20 mL
Total		500 mL

**Table:** Human stemMACS iPS-Brew XF medium (to keep at 4°C for up to 2 weeks)

Reagent	Final concentration	Amount
StemMACS iPS-Brew XF, Basal Medium		490 mL
StemMACS iPS-Brew XF, 50× Supplement	1×	10 mL
Total		500 mL

**Table:** MACS buffer (to keep at 4°C for up to 1 month)

Reagent	Final concentration	Amount
PBS		100 mL
Bovine serum albumin	0.5%	0.5 g
EDTA	2 mM	400 μL
**Total**		100 mL

## Step-by-step method details

### Peripheral blood mononuclear cells (PBMCs) isolation

**Timing: 1.5 h**

This section describes all the steps to isolate and freeze PBMCs from fresh blood.

1. Pour 10 mL of Ficoll-Paque PLUS in a 50 mL Faclon tube.
2. Pour 10 mL of the freshly drawn blood in another 50 mL Falcon tube and add 10 mL of PBS. Homogenize carefully the sample.

***Note:*** If the isolation cannot be performed right after blood drawn, the blood can be kept at room temperature for a maximum of 8 h on a rocker.

3. Carefully layer the diluted blood sample on Ficoll.

**CRITICAL:** When layering the sample do not mix it with Ficoll but slowly dispense it on top of the Ficoll volume.

4. Centrifuge at 750 g for 20 min at room temperature, with the brake off.

Remove as much of the plasma (upper yellow layer) as possible using a 10 mL pipette,

**CRITICAL:** Removing of the plasma should be done leaving the PBMCs layer (whitish ring) undisturbed at the interface between the plasma and the Ficoll.

5. Gently harvest the PBMC ring and transfer the cells in a new 50 mL Falcon: Add 30 mL of RPMI. Centrifuge at 550 g for 10 min at room temperature.
6. Discard the supernatant and resuspend the PBMCs in 10 mL of RPMI. Count the cells and centrifuge at 750 g for 10 min at room temperature.
7. Discard the supernatant and resuspend the cells in FBS/DMSO (90:10) at 10 million cells/mL. Distribute the cells in cryotubes between 5 to 10 million per cryotube and freeze at −80°C.

***Note:*** After 3 days at −80°C, cells can be transferred to liquid nitrogen for long-term storage.

### Erythroblast proliferation

**Timing: 10 days**

This section describes all the steps to obtain a culture composed at more than 90% of CD71+ CD36+ cells ready for reprogramming. This step of the protocol is based on a previous study by van den Akker et al. (van den Akker et al., 2010).

8. On day 0, thaw 10 million of frozen human PBMCs in PBS + FBS 20%.
  a. Centrifuge 5 min, 300 g at room temperature. Remove the supernatant.
  b. Resuspend the cells by performing up-and-downs with a pipette in 1.5 mL of Erythroblast medium.
  c. Transfer the cells into one well of a low-binding 24-well plate.
  d. Incubate cells at 37°C 5% CO_2_.
9. On day 1, add 500 μL/well of Erythroblast medium, gently resuspend the cells with a 1000 μL pipette to homogenize the culture and split into two wells (1 mL/well). Add another 500 μL of Erythroblast medium per well. Incubate the cells at 37°C 5% CO_2_.
10. On day 4, sort CD71+ cells by MACS. This procedure is performed using CD71 microbeads (Miltenyi) as described in the Manufacturer’s instructions: link. Briefly:
  a. Harvest all the cells and centrifuge 5 min at 300 g at room temperature.
  b. Discard the supernatant and resuspend the cells in 240 μL in MACS buffer. Add 60 μL of CD71 Miltenyi microbeads. Incubate for 15 min at 4°C.
  c. Add 6 mL of MACS buffer. Centrifuge 5 min at 300 g at room temperature. Discard the supernatant and resuspend the pellet in 500 μL of MACS buffer.
  d. Insert the MACS column into the MACS separator magnet. Wash column with 500 μL of MACS buffer. Add the 500 μL of CD71 labeled cells into the column. Wash with 3× 500 μL of MACS buffer.
  e. Remove the MACS column from the separator magnet and place it in a 15 mL Falcon collection tube. Add 1 mL of MACS buffer into the MACS column, immediately flush out positive fraction by firmly applying the plunger onto the column. Collect the flow through (CD71+ cells fraction). Centrifuge the positive fraction 5 min at 300 g.
11. Discard the supernatant and resuspend the pellet in 1 mL of Erythroblast medium (you should obtain about 500′000–1′000′000 cells). Transfer into a new well of a low-binding 24-well plate. Incubate cells at 37°C 5% CO_2_.

***Note:*** During the MACS procedure. Supernatant can be discarded by inverting the Falcon tube and cells are resuspended first by flicking the tube with a finger and then by up-and-down pipetting.

12. On day 5, resuspend erythroblasts by gentle flushing. Incubate cells at 37°C 5% CO_2_.
13. On day 6, if confluence is high (Figures 1A and 1B), resuspend the cells and split into two wells (500 μL/well). Add 1 mL/well of Erythroblast medium. If confluence is not high (Figures 1C and 1D), carefully remove 500 μL of medium (do not shake to plate, the cells must stay at the bottom of the well for medium removal) and add 1 mL of Erythroblast medium. Resuspend the cells to distribute them evenly in the well and incubate the cells at 37°C 5% CO_2_.

**Figure 1:**
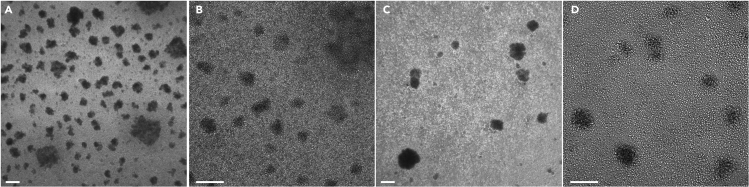
Confluence of erythroblasts post sorting (A and B) Pictures A and B (different zoom) show representative cultures of confluent erythroblasts forming many clusters. These cells need to be split into two wells on day 6. (C and D) Pictures C and D (different zoom) show representative cultures of non-confluent erythroblasts forming a few clusters that do not need to be split on day 6. Scale bar: 200 μm.
14. On day 8, repeat step 13.

**Figure 2:**
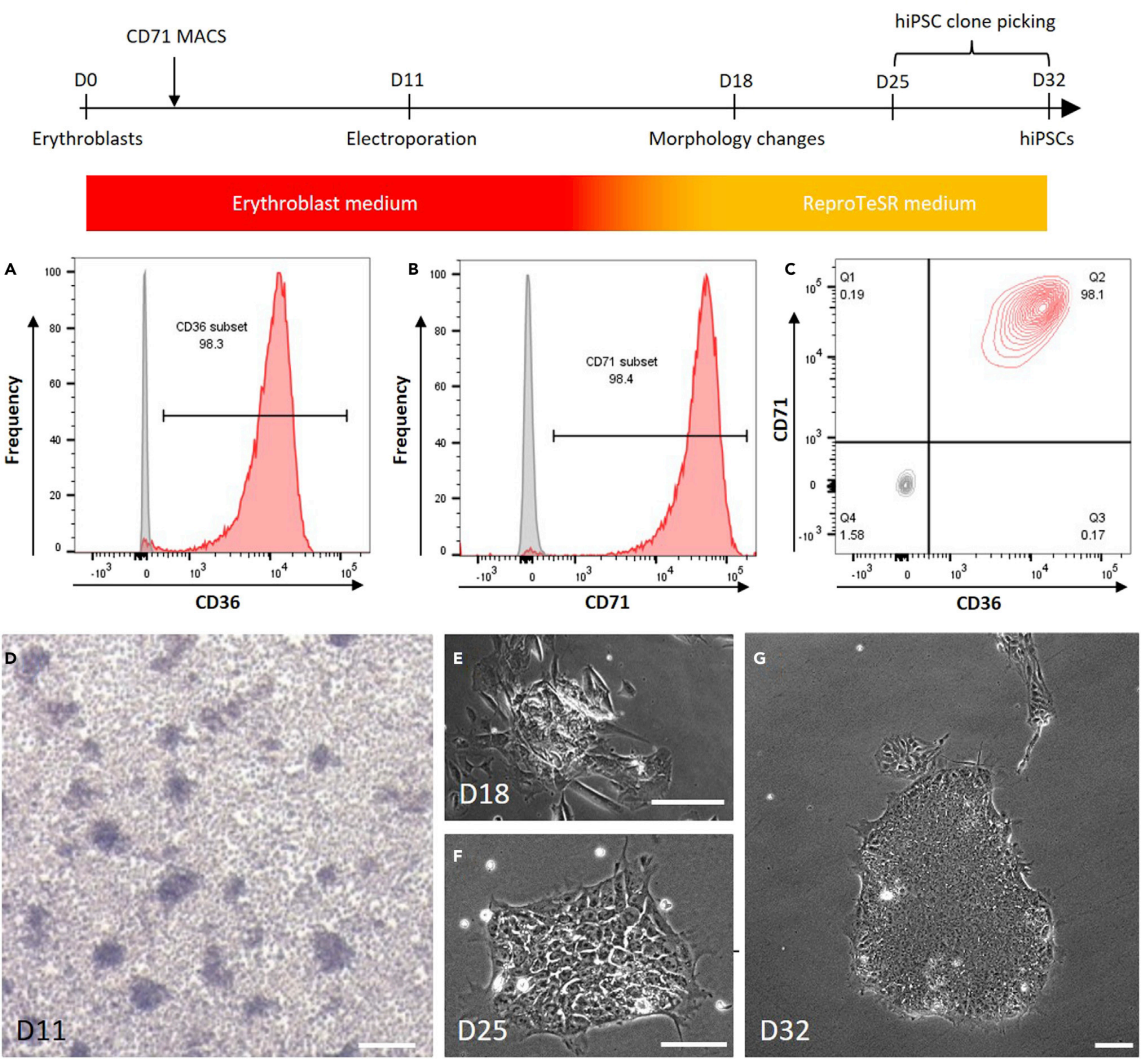
Timeline and representative pictures of hiPSC generation The timeline represents the general procedure to obtain hiPSCs from erythroblasts as well as the timing to obtain these cells. (A–C) Flow cytometry assessment of erythroblast culture before electroporation showing high purity (more than 98%) in CD71+ cells (A), CD36+ cells (B) with combined analysis of double positive cells (C). Red histograms and plot represent the stained cells while grey histograms and plot represent the unstained control. (D–G) Evolution of the morphology during cellular reprogramming showing erythroblasts culture (D – day 11), early reprogramming events leading to the generation of hiPSCs (E – day 18), an emerging hiPSC clone (F – day 25) and finally an established hiPSC clone (or colony) (G – day 32). Scale bars: 100 μm.

**CRITICAL:** By day 11, more than 90% of cells should be double positive for CD71 and CD36 and confluence should be good (Figure 2A). We routinely obtain more than 95% double positive cells but reprogramming can be carried on with a purity of 90% (troubleshooting 1). The enrichment of this population needs to be assessed by flow cytometry before proceeding to reprograming. Cells are resuspended by up and down pipetting, counted and around 150,000 cells are co-labeled with anti-CD71-PE and anti-CD36-FITC antibodies (standard flow cytometry staining procedure (Perriot et al., 2018)). Cells are then washed and analyzed by flow cytometry. While performing the purity check, erythroblasts should be kept in culture in the incubator. If percentage is lower than 90%, the culture can be prolonged in the same medium up to day 15 (troubleshooting 1).

### Erythroblast reprogramming

**Timing: 21 days**

This section describes the different steps to obtain hiPSCs from erythroblasts: electroporation of erythroblasts, culture until appearance of hiPSC clones and picking of these clones. See Figure 2 for a general timeline. These steps are based on the original publications by Chou et al. (Chou et al., 2011) and Okita et al. (Okita et al., 2011).

Step 15 directly follows the purity check by flow cytometry if the purity of erythroblasts is >90%.

15. On day 11, perform electroporation of erythroblasts with the episomes allowing cell reprogramming. Before starting the procedure, coat 3 wells of a 6-well plate with Matrigel.
  a. Prepare the reagents for electroporation:
    i Adjust the concentration of the plasmids pCXLE-hOCT3/4-shp53-F, pCXLE-hSK and pCXLE-hUL to 1 μg/μL.
    ii Mix the three plasmids together by adding 2 μL of each plasmid.
    iii Prepare the nucleofection mix using the solutions from the Human CD34+ Nucleofector kit (Lonza) as followed: 82 μL of nucleofection solution + 18 μL of supplement.
  b. Resuspend and count the erythroblasts.
  c. Collect 1 million erythroblasts and centrifuge 5 min at 300 g at room temperature. Completely remove the supernatant. Freeze the remaining erythroblasts as described in step 7 for PBMCs.
  d. Resuspend the cell pellet with the nucleofection mix (100 μL) and add the 6 μL of plasmid mix. Homogenize by up-and-down pipetting (avoid bubble/foam formation).
  e. Transfer cell suspension into the sterile cuvette provided with the Nucleofector kit and close the cuvette with the lid. The cell suspension must cover entirely the bottom of the cuvette without air bubble.
  f. Select the Nucleofector program T-016 (CD34+ cells program).
  g. Insert the cuvette into the Amaxa Nucleofector and start the T-016 program.
  h. Remove the cuvette. Foam must be visible on the surface of the mix indicating the electric shock happened.
16. Add 500 μL of erythroblast medium to the cuvette and transfer cell suspension into a 15 mL Falcon tube. Avoid repeated aspiration of the sample and bubble formation.
17. Use 500 μL of erythroblast medium to rinse the cuvette and add it to the cell suspension. Add 5 mL of erythroblast medium and distribute 2 mL of cell suspension in each Matrigel-coated well of the 6-well plate. Incubate at 37°C 5% CO_2_.
18. On day 13, carefully add 1 mL of erythroblast medium on the side of the well without removing medium from the plate not to disturb cells. Incubate at 37°C 5% CO_2_.
19. On day 14, carefully add 1 mL of ReproTeSR medium on the side of the well without removing medium from the plate not to disturb cells. Incubate at 37°C 5% CO_2_.
20. On day 16, repeat step 19.
21. On day 17, remove all the medium and slowly add 2 mL of ReproTeSR medium. Incubate at 37°C 5% CO_2_.

***Note:*** The erythroblasts frozen at this stage can be used for restarting the procedure in case of failure or to be used as reference for assessing cellular identity (see quality control section). Thawing of erythroblasts should be done as described in step 8.

**CRITICAL:** This medium change should be as gentle as possible to avoid detachment of erythroblasts from the plate. At this stage, some cells should start to adopt a morphology similar to epithelial or mesenchymal cells (Figure 2B) (troubleshooting 2).

22. From day 18 onward, change medium everyday as in step 21.

***Note:*** During the next seven days, reprogramming will continue to occur and newly generated hiPSCs will proliferate. Once hiPSCs are densely clustered together and form an identifiable colony (or clone) (Figures 2C and 2D), they can be individually picked to generate an independent hiPSC line (troubleshooting 2).

23. Human iPSC clones are picked between day 25 and 32 depending on the emergence of hiPSC colonies. Before starting hiPSC picking, coat one 35 mm dish with Matrigel for each clone to be picked. Once coating is done, remove the Matrigel solution.
  a. Add 2 mL of StemMACS iPS-Brew XF medium supplemented with Y-27632 (10 μM).
  b. Scrap the chosen hiPSC clone with a 200 μL micropipette and aspirate the clumps as you detach them.
  c. Transfer the clumps to a 35 mm dish.
  d. Name the newly generated hiPSC line with unique ID. Cells are now at passage 1.

***Note:*** Once a clone is picked and transferred to an individual 35 mm dish, it generates an independent clonal hiPSC line. Each hiPSC line should have a unique cell identifier. We suggest following the recommendations from the European Bank for induced Pluripotent Stem Cells (EBiSC) (Kurtz et al., 2018). The proposed format is as followed: XXXXXXi000-A.

“XXXXXX” (combination of 2–6 capital letters) for the origin of the line (institute or lab).
“i” for hiPSC lines or “e” for embryonic stem cell (ESC) lines.
“000” (numerical 3-digit code) for the donor ID.
“A” (capital letter) for the clone number.

24. Wash 3 times with PBS the well in which you just picked the hiPSC clone and add 1 mL of StemMACS iPS-Brew XF medium.
25. Repeat steps 16 and 17 individually as many times as needed to generate several lines.

**CRITICAL:** Human iPSC clones from one well of the 6-well plate should all be picked the same day to avoid picking twice the same clone.

**CRITICAL:** The picking procedure is to be done with extreme caution as you have to work over the wells without any antibiotics. Be very careful to avoid as much as possible passing over the wells in order to prevent from contamination.

***Note:*** Several hiPSC lines should be generated per donor to ensure that any phenotype observed in downstream experiments is the reflection of an intrinsic feature coming from the donor and not a byproduct of the reprogramming procedure that would be unique to a given hiPSC line regardless of the donor. Usually, at least two lines per donor are recommended when comparing groups of donors (e.g., patients vs healthy donors).

### Human iPSC amplification and cell banking

**Timing: 21 days**

This section describes how to amplify hiPSCs and bank them prior use for downstream experiments.

**Figure 3:**
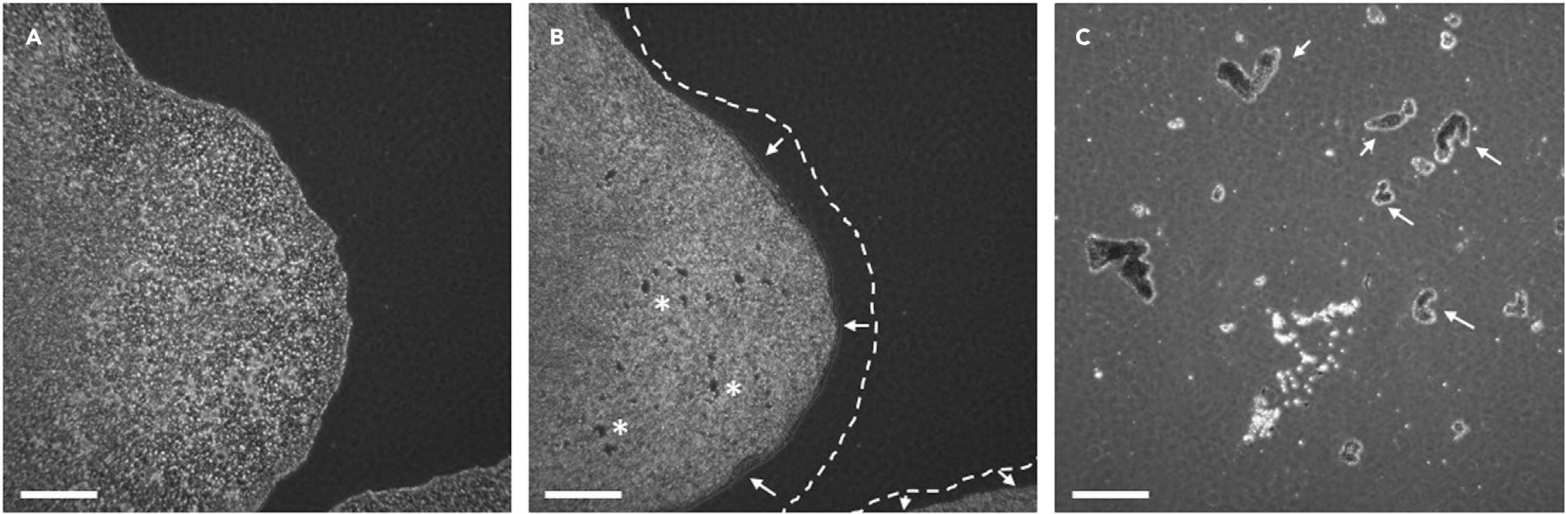
Representative pictures of hiPSC morphology during passaging (A) hiPSC colony before passaging. (B) hiPSC at the end of the incubation time (3–5 min) with the ReLeSR solution. Colonies exhibit retracted edges with refolding (arrows) as well as holes in the center (asterisks). (C) Detached hiPSC clumps (arrows) of the right size for plating (100–200 μm). Scale bar: 200 μm.
  d. Carefully remove ReLeSR solution from the dish.
  e. Add 1 mL of Complete StemMACS™ iPS-Brew medium and gently spill it on the cells. Perform a second gentle spilling on the cells. This procedure will result in detachment of hiPSCs from the plate and generates hiPSC clumps of 100–200 μm (Figure 3C).
  f. Harvest the cells in a Falcon tube and complete the volume of Complete StemMACS™ iPS-Brew medium to seed at a splitting ratio of 1:8 to 1:10 (2 mL of medium per dish).
  g. Plate the cells in 35 mm dishes and transfer the 35 mm dishes to the incubator (37°C, 5% CO_2_). Once in the incubator, move the dishes left to right and back and forth to ensure homogenous repartition of the clumps in the dish.
  h. Change the medium the day after passage and routinely as in step 27.

***Note:*** Some hiPSCs will not detach from the plate and will thus not be harvested. In particular, differentiated cells or colonies containing differentiated cells along with hiPSCs should not detach with this procedure. It is advised not trying to detach them, in order to keep a hiPSC culture as pure as possible, without differentiated cells.

26. The day following hiPSC picking, remove the medium of individual clones and add 2 mL of StemMACS iPS-Brew XF medium.
27. Repeat step 26 every day from Mondays to Thursdays. On Fridays, add 3.5 mL of StemMACS iPS-Brew XF medium instead of 2 mL. The medium does not need to be changed during the weekend.
28. Once the cells have reached about 80% confluence, passage them as followed:
  a. Remove the culture medium.
  b. Wash with 1 mL of PBS without Mg2+ Ca2+
  c. Add 1 mL of ReLeSR solution and incubate for about 3–5 min. Monitor the detachment process under the microscope (Figure 3). The edges of the colonies should have retracted and some holes formed within the colony (Figure 3B).

**CRITICAL:** The optimal seeding density should be determined empirically after several trials. A too high seeding density or poor clump repartition will result in higher differentiation. Usually, a 35 mm dish, which is confluent at 80% can be split up into 10 new dishes.

29. Cells should be amplified for at least three weeks to ensure the stabilization of the cell line and episome fading.

**Pause point:** Human iPSCs can be frozen at this point (troubleshooting 3). For logistic reasons, we recommend to select four lines per donor to move forward with quality controls and to freeze the remaining lines early as backup in case of quality control failures.

30. For freezing, harvest hiPSCs from one dish as described in step 28. a to e.
31. Harvest the entire clump suspension and centrifuge at 300 g for 5 min at room temperature.
32. Resuspend in 2 mL of Cryostor and divide them into four cryotubes, with 500 μL per cryotube.
33. Freeze at −80°C. After 3–7 days at −80°C, cells should be transferred to liquid nitrogen for long-term storage.
34. For cell thawing, quickly thaw the cell suspension at 37°C. The medium should not be completely thawed to increase cell survival.
35. Add 0.5 mL of StemMACS™ iPS-Brew medium to the vial.
36. Harvest the cells without up and down and transfer them into 4 mL of StemMACS™ iPS-Brew medium.
37. Centrifuge 5 min at 300 g at room temperature.
38. Remove the supernatant and resuspend the cell pellet in 4 mL of StemMACS™ iPS-Brew medium supplemented with Y-27632 (10 μM).
39. Distribute 2 mL per 35 mm Matrigel-coated dish and incubate at 37°C. The next day, change the medium and continue cell culture as described above.

### Human iPSC quality controls

**Timing: 3 weeks**

**Figure 4:**
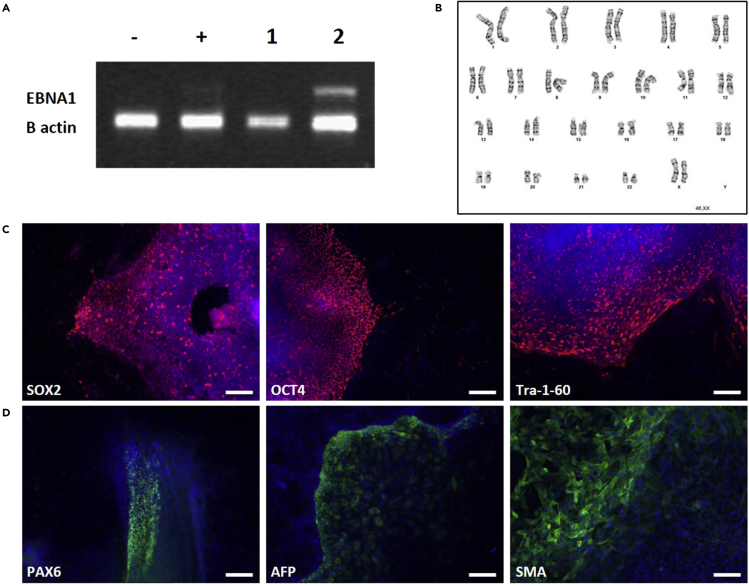
Quality controls to ensure the usability of hiPSCs (A) Assessment of episome disappearance in hiPSCs. From left to right: Negative ctrl (-), Limit of detection sample (+), hiPSCs that lost the episome (1), hiPSCs that did not lose the episome (2). (B) Representative picture of a normal hiPSC karyotype showing that no abnormality was acquired during the reprogramming phase. (C) Full pluripotency was monitored by immunofluoresence staining of pluripotency markers (SOX2, OCT4, Tra-1-60) (in red). Cells were counterstained with DAPI (in blue). Scale bars: 200 μm. Pictures were acquired with inverted microscope Axiovision Observer.Z1. (D) After differentiation of hiPSC into embryoid bodies (EB), the capacity of hiPSC to form the three embryonic germ layers was assessed by IF staining with one marker per germ layer (green): ectoderm (PAX6), endoderm (AFP) and mesoderm (SMA). Cells were counterstained with DAPI (blue). Scale bars: 200 μm.

Prior to downstream use, hiPSCs need to pass several quality controls (QCs) (Figure 4). These controls are part of the good practices for hiPSC research as described by the EBiSC (Steeg et al., 2021; Sullivan et al., 2018). These QCs are absolutely necessary to ensure of the good quality of the hiPSCs. They have been regrouped in Table 1.

**Table 1:** Summary of QCs to be performed before using hiPSC for downstream experiments

QC	Method	Sample type	Kit	Instructions	Frequency
Negative for bacteria, yeast, fungi infection	Visual inspection of antibiotic-free cultures	Live cell culture	N/A	N/A	Every day
Negative for mycoplasma infection	Enzymatic	Cell culture supernatant	MycoAlertTM Mycoplasma Detection Kit	Link	Every 10 passages
Negative for viral infection (HIV, HBV, HCV)	PCR	On donor somatic cells prior to hiPSC reprogramming	N/A	To be done by a clinical lab at enrollment of the donor	Once
Cellular identity	Short tandem Repeat allele profile	Cells	Promega PowerPlex® 16 System	Link	At cell banking
Genomic stability	G-banding karyotyping	Cells	N/A	N/A	Every 10 passages
Morphology	Visual inspection	Live cell culture	N/A	N/A	Every day
Clearing of episomes	PCR	Extracted DNA	Your favorite PCR kit + primers (see Table 2)	N/A	At cell banking
Pluripotent marker expression	Immunocytochemistry on Oct4, Tra-1-60 and Sox2	Fixed cells	N/A (see key resources table for antibodies)	Standard IF staining procedure (Perriot et al., 2018)	Once
Pluripotency	Embryoid body formation and IF staining for Pax6, AFP and SMA	Fixed cells	N/A (see key resources table for antibodies)	See below	Once

**Table 2:** Primer sequences for assessing clearance of EBV-based episomes

Primer	Sequence
EBNA1_forward	AGA TGA CCC AGG AGA AGG CCC AAG C
EBNA1_reverse	CAA AGG GGA GAC GAC TCA ATG GTG T
β-Actin_forward	CAT GTA CGT TGC TAT CCA GGC
β-Actin_reverse	CTC CTT AAT GTC ACG CAC GAT

To generate embryoid bodies in order to assess the pluripotency of hiPSCs, follow the procedure below.

40. Remove hiPSC medium from one 35 mm dish at 80% confluence.
41. Add 1 mL of accutase.
42. Incubate until the edges of the hiPSC colonies start retracting (similar to Figure 3B).
43. Remove accutase and add 2 mL of DMEM/F-12 medium supplemented with 20% fetal bovine serum (FBS).
44. Carefully scrap the hiPSC colonies using a cell lifter taking care of keeping the cells as clumps.
45. Transfer the hiPSC clumps into 1 well of a low-binding 6-well plate.
46. The next day, EB should have formed. Split them into 2 wells of a low-binding 6-well plate and add 1 mL of DMEM/F-12 + 20% FBS to each well.
47. Perform 2/3 of medium change every 2–3 days for 8 days.
48. On day 10 after EB formation, plate the EBs on Matrigel. At this stage, EB should have a round or oblong morphology with size between 300–600 μm.
49. Continue the culture by changing the medium every 2–3 days.
50. After 14 days, fix the cells with PFA 4% for 10 min at room temperature. Wash 3 times with PBS and stain the EBs for assessing the expression of the markers from the three embryonic germ layers (ectoderm (Pax6), mesoderm (SMA) and endoderm (AFP)).

## Expected outcomes

The protocol described here allows obtaining between 10 to 20 hiPSC clones per donor. In addition to the protocol to generate the hiPSCs, we provide the methods to operate the necessary QCs ensuring that the hiPSCs obtained classify for downstream experiments according to the EBiSC standard. Not all clones generated need to be assessed for QCs but all clones used in any study should passed all mandatory QCs. Overall, this manuscript contains all the information to obtain fully characterized hiPSC clones free of transgenes and without BCR/TCR recombination, from a single blood draw.

## Limitations

The protocol detailed here reproducibly generates hiPSCs responding to EBiSC standards, using homemade media and thus less expensive than using commercial kits. One limitation is that the protocol may not be directly transposable to generate hiPSC from fibroblasts since it starts from erythroblasts. It mostly is an advantage for institutes that have the capacity to recruit volunteers for their research given that a blood draw is easier to perform and less invasive than a skin biopsy. However, centers that cannot recruit their own volunteers need to rely on cell repositories. Since these cell banks mostly lack isolated PBMCs and usually bank fibroblasts, it may represent an obstacle for direct use of this protocol.

## Troubleshooting

### Problem 1

Low erythroblast purity (step 14).

Erythroblasts (double positive CD71+CD36+) should represent more than 90% of the cell culture. A lower proportion indicates issues in the erythroblasts amplification and may impact negatively the formation of hiPSCs. Most likely, poor erythroblast purity arise from issues during the amplification phase which may be due to two main reasons. First, poor storage of cytokines, growth factors and media. Second, too high or too low cell confluence will decrease erythroblast amplification.

### Potential solution

The issue may be avoided by following some recommendations. First, to strictly respecting expiration date and storage conditions indicated by the supplier for each reagent. The complete erythroblast medium should not be kept more than a week and stored at 4°C at all time. We also recommend limiting at the maximum keeping the medium at room temperature. During culture, avoid at all cost to let the medium to turn yellow-orange.

If a low proportion of erythroblast is still obtained despite these recommendations, it is possible to further pursue the culture in the same conditions for four additional days and to reassess CD71 and CD36 expression at day 15. The proportion of erythroblasts should be good at this point.

### Problem 2

Absence of cellular reprogramming (step 23).

If no changes of morphology are observed by day 24, it is to be considered that the reprogramming failed.

### Potential solution

Failure in reprogramming is mostly associated with the quality of the erythroblasts or with an error in the electroporation (step 15). Before starting the electroporation, ensure that the erythroblasts are pure (see problem 1 above) and that the viability is more than 85%. Potential errors at the electroporation stage include miscalculation of the plasmids concentration, errors during mix preparation or technical error using the electroporation device. Make sure that all the parameters above are correct. An initial assessment of electroporation efficacy may be performed using a GFP plasmid. We recommend using the pCXLE-EGFP plasmid. Last, make sure to work quickly and to put back electroporated erythroblasts quickly in culture. Any delay causes cell death which may result in failure of reprogramming.

### Problem 3

Poor hiPSC survival after thawing (step 34).

The survival of hiPSCs post thawing is low and does not allow recovering the hiPSC line.

### Potential solution

Most likely, the freezing steps were not executed properly. In particular, one should avoid breaking hiPSC clumps as much as possible and work as fast as possible to ensure proper cell recovery. Conversely, the thawing procedure should also be performed as fast as possible and avoiding breaking hiPSC clumps. One key element is to minimize the number of up and down done pipetting the cell suspension. Omission of Y-27632 at thawing will also result in lower cell survival.

## Resource availability

### Lead contact

Further information and requests for resources should be directed to the lead contact, Renaud Du Pasquier, renaud.du-pasquier@chuv.ch.

### Materials availability

This study did not generate any unique reagents.

## Data Availability

This study did not generate any unique datasets or code.
